# Input Shaping Based on an Experimental Transfer Function for an Electrostatic Microscanner in a Quasistatic Mode

**DOI:** 10.3390/mi10040217

**Published:** 2019-03-27

**Authors:** Kwanghyun Kim, Seunghwan Moon, Jinhwan Kim, Yangkyu Park, Jong-Hyun Lee

**Affiliations:** 1 School of Mechanical Engineering, Gwangju Institute of Science and Technology, Gwangju 61005, Korea; khinmf13@gist.ac.kr (K.K.); goalermen2@gist.ac.kr (J.K.); yangkyu.park1@ucalgary.ca (Y.P.); 2 WeMEMS Co., Gwangju 61005, Korea; wemems7.msh@gmail.com

**Keywords:** microscanner, input shaping, open-loop control, quasistatic actuation, residual oscillation, usable scan range, higher-order modes

## Abstract

This paper describes an input shaping method based on an experimental transfer function to effectively obtain a desired scan output for an electrostatic microscanner driven in a quasistatic mode. This method features possible driving extended to a higher frequency, whereas the conventional control needs dynamic modeling and is still ineffective in mitigating harmonics, sub-resonances, and/or higher modes. The performance of the input shaping was experimentally evaluated in terms of the usable scan range (USR), and its application limits were examined with respect to the optical scan angle and frequency. The experimental results showed that the usable scan range is as wide as 96% for a total optical scan angle (total OSA) of up to 9° when the criterion for scan line error is 1.5%. The usable scan ranges were degraded for larger total optical scan angles because of the nonlinear electrostatic torque with respect to the driving voltage. The usable scan range was 90% or higher for most frequencies up to 160 Hz and was drastically decreased for the higher driving frequency because fewer harmonics are included in the input shaping process. Conclusively, the proposed method was experimentally confirmed to show good performance in view of its simplicity and its operable range, quantitatively compared with that of the conventional control.

## 1. Introduction

MEMS (microelectromechanical systems) optical scanners are widely used micro systems, each of which is capable of driving a single mirror in two axes. In particular, the electrostatic actuation approach has advantages in terms of its size, power consumption, and integration and is therefore widely applied to such fields as optical coherence tomography (OCT) [1], laser projection display [2], and light detection and ranging (LiDAR) [3]. In these imaging systems, the usable scan range (USR), one of the important performance parameters of the scanner, depends on the uniformity of the beam steering speed. Here, the USR in percentiles, defined as the ratio of the usable range that satisfies a certain criterion (for example, the maximum deviation from a desired scan trajectory), will be used as one of the performance indices in input shaping.

**Figure 1:**
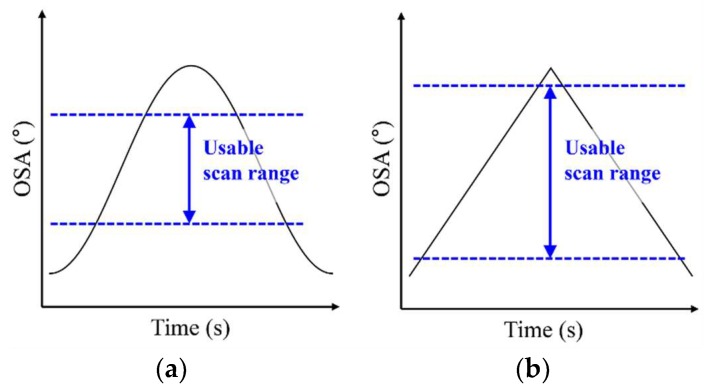
Definition of usable scan ranges (USRs) of the optical scan angle (OSA) for different waveforms: (**a**) sinusoidal and (**b**) triangular.

Figure 1a,b show the speed uniformity of the optical scan angle (OSA) for two types of waveform in the scan outputs. In terms of the USR, the triangular waveform has advantages over the sinusoidal waveform in that there is no need for post-processing to enhance the uniformity of the images [4]. Thus, a triangular scan is generally preferred to a sinusoidal scan for high-quality images.

However, the input voltage in a triangular waveform may cause serious residual oscillation, resulting in distortions of the imaging [5] if one of the harmonics of the input frequency is close to or matched by the fundamental torsional mode of the microscanner. To solve this problem, studies have been performed on input shaping, such as open-loop control [6,7] and closed-loop control [8,9].

In the case of open-loop control, mechanical parameters such as the moment of inertia, damping coefficient, and torsional spring constant are extracted for a second-order dynamic model based on the measured frequency response of the microscanner. This model is used for inverse filtering to eliminate residual oscillation from a desirable arbitrary scan output [10,11].

However, the second-order transfer function as a dynamic model cannot accurately represent the spectral data, even for the fundamental mode, when the scanner has nonlinearity in the drive characteristics. As a result, distortion may appear on the waveform of the scan output, even if input shaping is performed for the given microscanner. Another disadvantage is that residual oscillation cannot be avoided if the higher modes and/or sub-resonance in the output transfer function are not negligible [12].

In the case of closed-loop control, the angular position of the mirror can be accurately adjusted by feeding back the positioning error to the controller. The angular position of the mirror can be further corrected by the additional use of the second-order transfer function which was already extracted from the open-loop control [3,9]. Consequently, closed-loop control shows good performance with high robustness regardless of the residual oscillations or external disturbance. Moreover, closed-loop control can maintain good performance despite the presence of a fatal error in the second-order dynamic modeling. However, this closed-loop control approach requires position detection devices, such as capacitive [13,14] and piezoresistive [15] sensors, making the fabrication process more complicated.

In this paper, we examine an input shaping method based on an experimental transfer function (ISETF) to effectively obtain the desired output. Unlike conventional open-loop control using a modeled transfer function, this method directly uses the experimental transfer function (ETF) with no dynamic modeling of the microscanner. The ISETF makes use of the ETF including harmonics, higher modes, and/or sub-resonance so that the transfer function can be obtained more accurately than through conventional open-loop control.

This paper is organized as follows. In Section 2, the experimental conditions, including the comb structures of the electrostatic microscanner and linearization between the driving voltage and electrostatic torque, are described. Undesired oscillation induced by the resonance frequency is also exemplified for the microscanner under testing. In Section 3, the procedure to obtain the ETF is presented. In Section 4, to quantitatively validate the method, a deviation from a desired scan output is evaluated using an electrostatic microscanner driven in a quasistatic mode. Using the variance data, the limitations of the proposed ISETF are examined with respect to OSA and frequency, and the performance of the ISETF is compared to those of other open-loop and closed-loop controls. Finally, the conclusions of this study are given in Section 5.

## 2. Quasistatic Microscanner

### 2.1. Characteristics of the Microscanner

**Figure 2:**
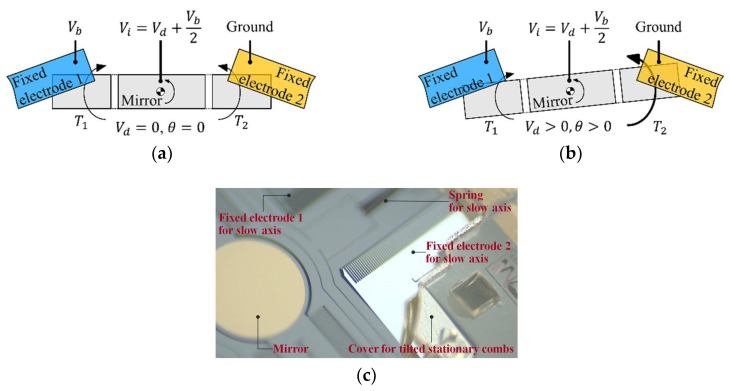
Linearization between electrostatic torque and driving voltage (V-linearization) for the slow axis: (**a**) initial position (*V_d_* = 0), (**b**) rotated position (*V_d_* > 0), and (**c**) photograph of the microscanner with tilted stationary combs.

The device used in this study is the two-axis electrostatic microscanner that was previously reported by our group [16]. As shown in Figure 2a, the fixed comb electrodes in the slow axis were tilted for quasistatic actuation, where linearization with driving voltage was conducted using a pair of comb actuators. Only the actuator in the slow axis was tested to quantitatively validate the proposed ISETF, because input shaping can be basically applied to the scanner operated in a quasistatic mode. Details of the dimensions of the electrodes are listed in Table 1.

### 2.2. Linearized Actuation

An electrostatic microscanner has the inherent nonlinearities of the electrostatic torque between two electrodes being proportional to the square of the driving voltages. The electrostatic torque can be linearized with respect to the driving voltage in the case where one pair of the electrostatic actuators is driven independently [17]. This voltage linearization (so-called V-linearization) allows the ISETF to be applicable to the microscanner because the ISETF is based on a linear transfer function.

For the V-linearization of the microscanner, the DC bias voltage (*V_b_*), the ground, and the input voltage (*V_i_*) are applied to fixed electrode 1, fixed electrode 2, and the mirror, respectively, as shown in Figure 2a, where *V_i_* is the sum of the driving voltage (*V_d_*) and *V_b_*/2. The electrostatic torques (*T*_1_, *T*_2_) generated on movable electrodes 1 and 2 can be calculated using Equations (1) and (2), respectively. When *V_d_* is zero, the magnitudes of *T*_1_ and *T*_2_ are equal to each other, staying at the initial position. Figure 2b shows that the value of *V_d_* is greater than 0, resulting in the rotation of the mirror in the counterclockwise (positive sign) direction. Note that the total torque (*T*_1_ + *T*_2_) is linearly proportional to *V_d_*, as expressed in Equation (3). Likewise, the linearization procedure can be extended to the case where the scanner is driven by alternating current (AC) driving voltage. Figure 2c shows a photograph of the microscanner with the tilted stationary combs used in this study.

**Figure 3:**
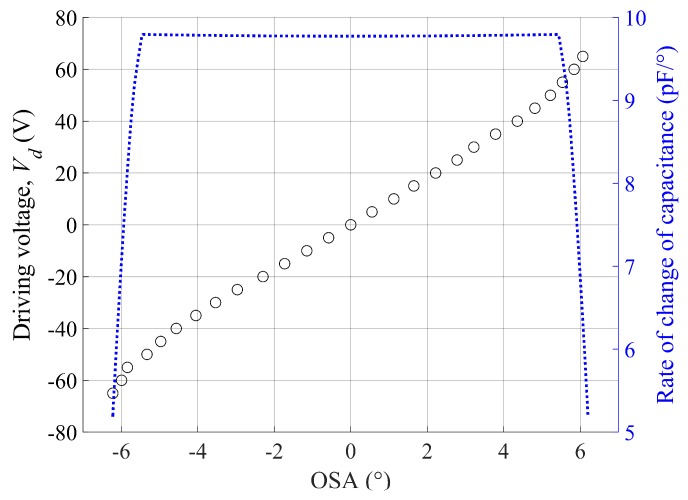
Nonlinear characteristics of the microscanner in quasistatic mode under the condition of V-linearization (open circles: experimental OSA; dotted line: calculated rate of change of capacitance).

The experimental OSA under the V-linearization condition is shown with respect to the direct current (DC) voltage in Figure 3, where the bias voltage, *V_b_*, is 130 V, and the driving voltage, *V_d_*, is in the range from −65 V to 65 V. Despite the V-linearization process, nonlinearity is still witnessed when the mirror is rotated beyond a certain critical angle. This result is believed to be mainly determined by the change rate of the overlapped area between the pair of comb electrodes.

Moreover, Equation (3) shows that the electrostatic torque is also proportional to the rate of change of the capacitance. To determine the cause of the nonlinearity witnessed at a large scan angle, the rate was calculated with respect to the OSA, as shown in Figure 3. The calculation result indicates that the linearity of the rate is smaller than 1% in the OSA range between −5° and 5°, whereas the rate of change of capacitance drastically decreases outside the linear range; i.e., the so-called C-nonlinearity. This supports the observation that the nonlinearity of OSA with respect to the input voltage is mainly caused by C-nonlinearity. The ISETF will be examined over the OSA range up to ±6.5° (equivalently, 13° in total OSA) to find its application limit in Section 4.1. The scan angle can be extended beyond the linear range if a new ETF is extracted at that scan angle, as long as the scan output remains periodic in a steady state.

(1) $$T_{1}=\frac{1}{2}\frac{\partial C}{\partial \theta }{\left(V_{d}-\frac{V_{b}}{2}\right)}^{2}$$

(2) $$T_{2}=\frac{1}{2}\frac{\partial C}{\partial \theta }{\left(V_{d}+\frac{V_{b}}{2}\right)}^{2}$$

(3) $$T=-T_{1}+T_{2}=V_{b}V_{d}\frac{\partial C}{\partial \theta }$$

### 2.3. Residual Oscillation

**Figure 4:**
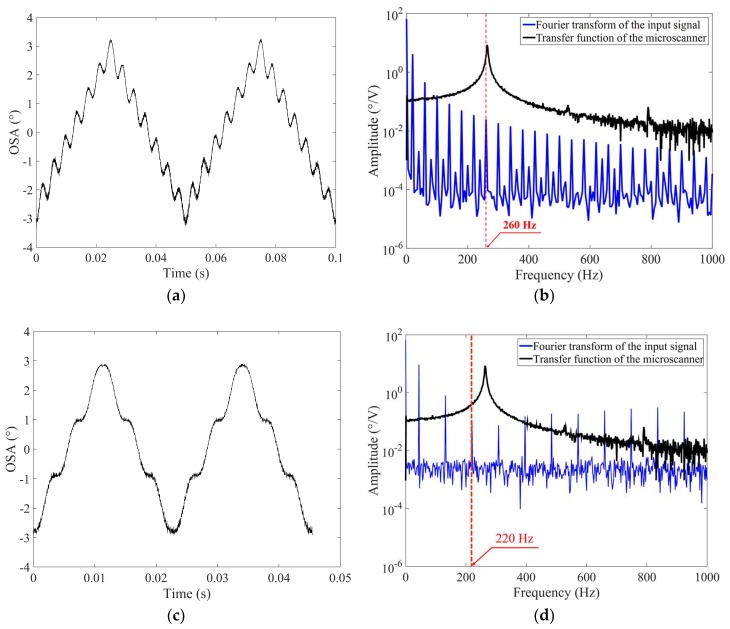
Exemplified dynamic response of the fabricated microscanner driven by a triangular input voltage at 20 Hz (*V_d_*: 25 V): (**a**) scan output with severe residual oscillations in the time response and (**b**) Fourier-transformed input signal (blue) and transfer function of the microscanner (black), (**c**) scan output at 44 Hz with fewer residual oscillations, and (**d**) Fourier-transformed input signal at 44 Hz (blue) and transfer function of the microscanner (black).

When a triangular input voltage is provided, undesired residual oscillations can occur in the scan output. Figure 4a shows the experimental scan output with severe residual oscillations in a steady-state response when a triangular input waveform is applied at a driving frequency of 20 Hz to the microscanner. The driving frequency of 20 Hz was selected as an example, showing that this residual oscillation occurs when one of the harmonics of the input frequency might be near to the frequency of the fundamental mode in the scanner. 

Figure 4b shows the Fourier-transformed input voltage and the transfer function of the microscanner, where the sixth harmonic (260 Hz) is very close to the torsional mode at 264 Hz (the experimental procedure to obtain the transfer function will be described in detail in Section 3.1). Thus, an undesired residual oscillation of 260 Hz appears in the scan output, despite the amplitude of the sixth harmonic (260 Hz) being relatively small. Thus, the ISETF was proposed in this paper to obtain a desired output waveform by effectively reducing undesired residual oscillation. Figure 4c shows the experimental scan output at 44 Hz when the ISETF is not yet applied to the input. The residual oscillation at 44 Hz was less serious than that at 20 Hz because the harmonics of the driving frequency 44 Hz are far from the frequency of the fundamental mode, as shown in Figure 4d, compared with that of 20 Hz. The application of the proposed ISETF is not limited to these frequencies but will be evaluated for the frequency range up to 300 Hz to find its application limit in Section 4.2.

## 3. ISETF Procedure

### 3.1. Experimental Conditions

The relationship between voltage input, *X*(*f*), and scan output, *Y*(*f*), can be defined by a transfer function *H*(*f*), as expressed in Equation (4):

(4) $$H(f)=\frac{Y(f)}{X(f)},$$

where *f* is the driving frequency. The ETF of the microscanner can be obtained using the impulse input or the frequency sweep. The impulse response method is advantageous in that the transfer function is simply obtained by applying a single pulse input. The pulse width should be small to include a sufficient number of high-frequency components. Meanwhile, the frequency sweep method can obtain an accurate transfer function for the high-frequency region. However, this method has several disadvantages related to being a complicated and time-consuming process because the experiment requires waiting at each measurement frequency until the steady-state response is obtained.

In this study, the impulse response method was employed to evaluate the ISETF with a pulse width of 500 μs or, equivalently, a frequency bandwidth of 2 kHz. The frequency bandwidth is large enough to include at least six frequency components for driving frequencies up to 300 Hz, over which the input shaping calculations are performed. Note that 300 Hz is higher than generally required for the scan frequency of the slow axis and is also higher than the frequency of the fundamental mode (264 Hz) of the scanner, which needs to be compensated for in most input shaping methods. The sampling rate for the ISETF was 50 kHz, which is sufficiently beyond the Nyquist criterion to provide an accurate measurement for the aforementioned driving conditions. The sampling time was set to one second, indicating that the frequency interval is 1 Hz.

It is also important that the response of the microscanner to the input pulse should remain in the linear region. Thus, in the experiment using impulse input, the magnitude of the OSA should be smaller than 5°, or, equivalently, the amplitude of the impulse voltage should be smaller than 55 V (refer to Figure 3).

### 3.2. Experimental Setup

**Figure 5:**
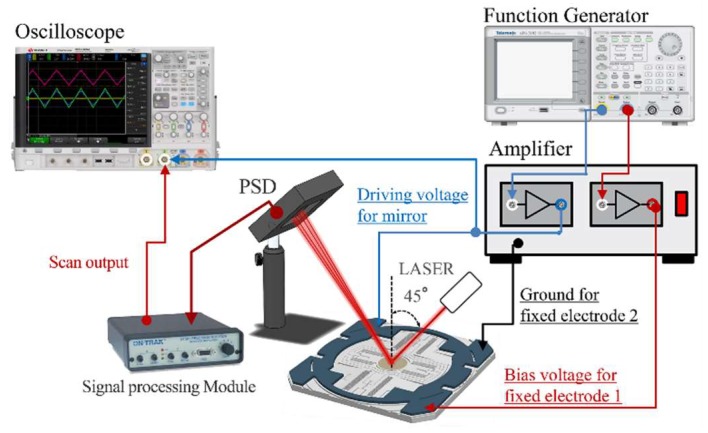
Experimental setup for obtaining a transfer function of the microscanner.

The experimental setup to obtain the transfer function is shown in Figure 5, where the laser diode emits a laser beam (wavelength = 633 nm) through the collimator (Pigtailed collimator, OZ Optics, Ottawa, ON, Canada) to the mirror with an incident angle of 45°. As the first step of the experiment, the input pulse is applied to the scanner through a two-channel function generator (AFG3102, Tektronix, Beaverton, OR, USA) with a bandwidth of 240 MHz and a power amplifier (A400D, FLC electronics, Partille, Sweden) with a bandwidth of 500 kHz, consecutively. Next, a position-sensitive detector (PSD) (PSM2-45, Ontrak Photonics, Irvine, CA, USA) measures the position of the laser/beam reflected from the oscillating micromirror. Finally, the PSD signal is digitized at 50 kHz using an oscilloscope (DSO-X-4024A, Keysight, Santa Rosa, CA, USA) over a period of one second to extract 50,000 data points in total.

### 3.3. Extraction of the Experimental Transfer Function

**Figure 6:**
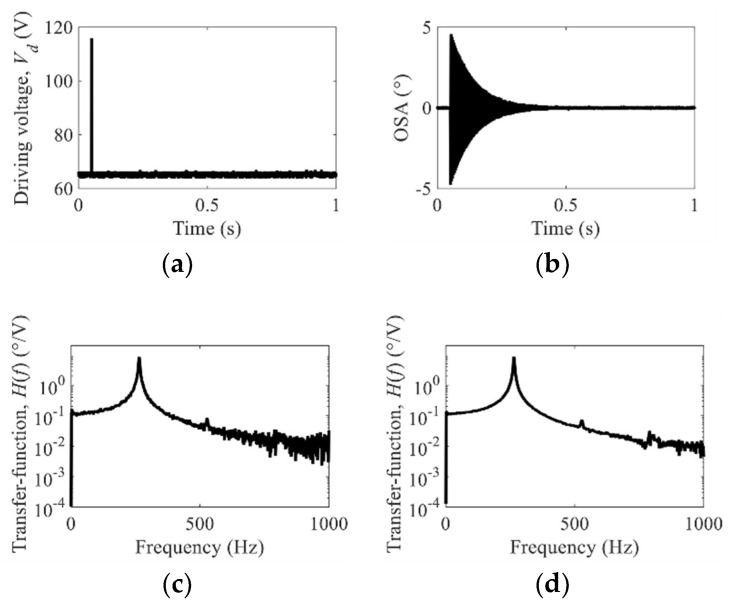
Estimation process of the experimental transfer function (ETF) for input shaping: (**a**) input impulse in the time domain (*V_d_*: 55 V, *V_b_*/2: 65 V, pulse width 500 μs), (**b**) output response in the time domain, (**c**) an ETF, and (**d**) a ten-times averaged ETF revealing the second and third harmonics.

Figure 6a,b represent the measured pulse input and the scan output of the mirror, respectively; these data were used to estimate the ETF through Equation (4), as shown in Figure 6c. The ETFs were averaged ten times to reduce the noise, as shown in Figure 6d. We believe that the effect of averaging on the performance of the control system lies in the effective reduction of the pulse width in the impulse response; thereby, the averaged data more clearly reveal that harmonics exist at 527 Hz and 790 Hz.

Unfortunately, a conventional open-loop control that makes use of the second-order dynamic modeling can only account for the fundamental mode. On the contrary, the proposed ISETF method also has the potential to compensate for the harmonics of the fundamental mode, higher resonance modes, and sub-resonances. Nevertheless, the higher-order modes were not observed in the experiments because their frequencies exist beyond the effective bandwidth (2000 Hz), as determined by the pulse width of the input impulse. The sub-resonances, which arise from non-linear actuation forces proportional to the square of the driving voltage, were also eliminated by V-linearization, as described in Section 2.2.

### 3.4. Input Shaping for Triangular Output

A triangular waveform is one of the most desirable output forms, *d*(*t*), in view of the USR. The ISETF method was employed to verify the effectiveness of input shaping to achieve the ideal triangular output. The shaped input signal in the time domain, *v*(*t*), used to generate *d*(*t*), can be calculated using the ETF obtained in Section 3.3 as follows:

(1) The ideal triangular output, *d*(*t*), is Fourier-transformed to obtain *D*(*f*) in the frequency domain by using Matlab software.
(2) *D*(*f*) is divided by *H*(*f*) to acquire the input signal in the frequency domain, *V*(*f*), as shown in Equation (5).
(3) The shaped input signal in the time domain, *v*(*t*), is obtained through inverse fast Fourier transform (inverse FFT).
(4) The calculated shaped input signal in the time domain, *v*(*t*), is converted to the filename extension ‘.tfw’ using ArbExpress Application software so that it can be recognized in the function generator.
(5) The converted shaped input signal is stored in the function generator through a universal serial bus (USB) memory so that it can be applied to the microscanner.
  (5) $$V(f)=\frac{D(f)}{H(f)}$$

**Figure 7:**
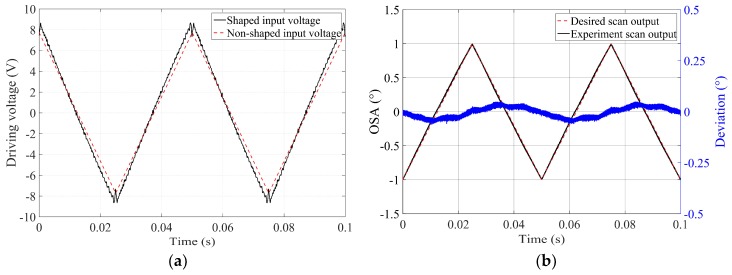
Performance of the input shaping method based on an experimental transfer function (ISETF): (**a**) the shaped input and non-shaped input for the optical scan range from −1° to 1° and (**b**) comparison between the ideal triangular output and the experimental scan output obtained using the shaped input based on an experimental transfer function (ETF).

To examine the performance of the ISETF described above, the input shaping was conducted for an ideal triangular output at 20 Hz with an optical scan range from −1° to 1°. Note that the scan angle is within the linear range of the microscanner, as shown in Figure 3. The shaped input signal (black line) of the ISETF produces negligible overshoots (11%) at the driving voltage, which should not put strain on the driver electronics, as shown in Figure 7a. The experimental scan output of the slow axis (black line) was directly compared with the ideal triangular output (dashed line in red) to easily quantify the deviation error (blue line), as shown in Figure 7b. The undesired residual oscillation due to the fundamental mode of the scanner was drastically diminished so that the scan output very closely approaches the ideal triangular output. Meanwhile, the model-based approach can produce significant noise in the high frequency range of the scan output. The noise was effectively removed by applying a low-pass filter, still leaving considerable residual oscillations at the harmonic frequencies of the shaped input. The scan phases were not apparently affected by the ISETF, and a quantitative evaluation of the proposed ISETF will be discussed in terms of the USR in Section 4.1.

## 4. Application Limits of ISETF

### 4.1. Optical Scan Angle (OSA)

**Figure 8:**
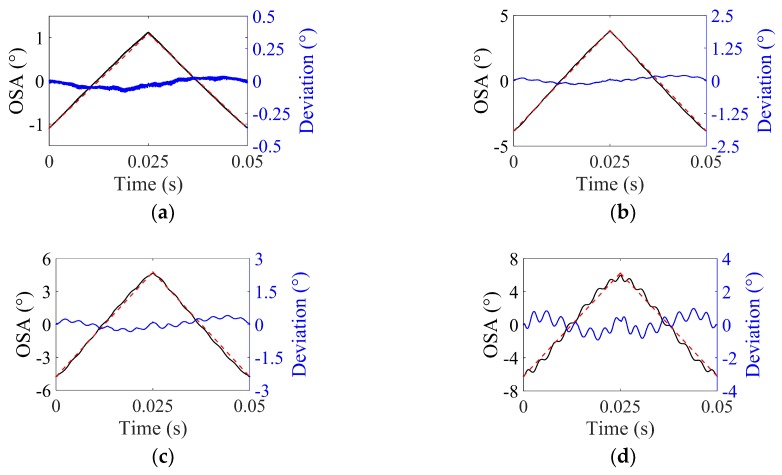
Performance of the ISETF with respect to the driving voltage, *V_d_* (driving frequency: 20 Hz, sampling rate: 50 kHz): (**a**) *V_d_* = 10 V, (**b**) *V_d_* = 35 V, (**c**) *V_d_* = 45 V, and (**d**) *V_d_* = 65 V (scan output using ISETF: solid line in black; ideal triangular output: dashed lines in red).

As mentioned in Section 2.2, to examine the application limits of ISETF due to C-nonlinearity, an input shaping process was experimentally implemented for total optical scan angles (total OSAs) from 2° to 12° at the driving frequency of 20 Hz. Figure 8 shows a comparison between the experimental scan output (black line) and the ideal triangular output (dashed lines in red). As the driving voltage increases, the scan output gradually differs from the ideal triangular output. More specifically, the residual oscillation is negligible up to 35 V and becomes serious at 65 V. The residual oscillation at higher angles (total OSA 13°), where the ISETF method becomes insufficient to suppress them, still contains the fundamental mode rather than other higher modes.

**Figure 9:**
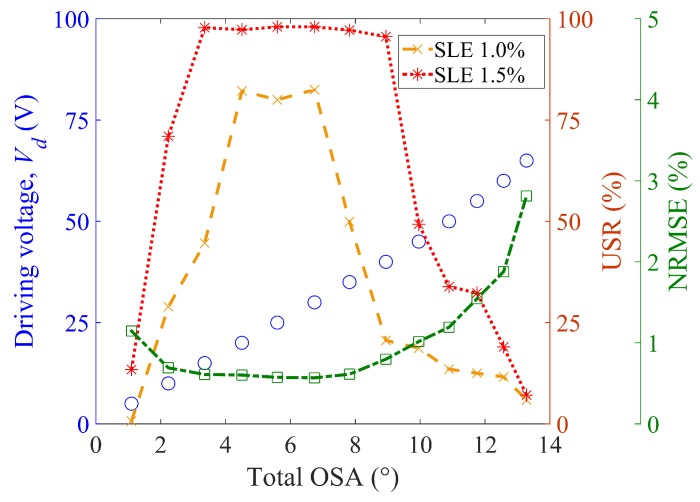
Experimental performance of the proposed input shaping with respect to the total optical scan angle (total OSA) (driving frequency: 20 Hz, sampling rate: 50 kHz): driving voltage with respect to total optical scan angle (circles in blue), usable scan range (dashed lines), and normalized root-mean-square error (NRMSE) (dash-dotted line in green).

For statistical evaluation, the root-mean-square error was calculated with reference to the ideal triangular output. Next, the calculated value was divided by the total OSA to obtain a normalized root-mean-square error (NRMSE), as a percentile, as shown in Figure 9. The NRMSE value remains at a sufficiently low level (1% or smaller) within the linear region, as confirmed in Figure 3. The NRMSE, however, shows the tendency to abruptly degrade beyond the linear region because the nonlinearity of the electrostatic torque becomes more serious for larger total optical scan angles (or driving voltages). The potential application of this method includes LiDAR, which requires around 100 lines for the slow axis, although a 1% scan accuracy is not nearly precise enough for general display systems.

As a strict performance index, the USR was also estimated under a given criterion defined by the scan line error (SLE) as a percentile. For example, an SLE of 1% indicates that a certain scan line deviates from the intended trajectory by 1% of the total OSA. In other words, the USR is the longest scan range where the difference between the desired output and the scan output is less than 1% SLE. The estimated USR is shown in Figure 9 with respect to the total OSA at for SLEs of 1.0% and 1.5%.

When the SLE is 1.5%, the USR is 96% or higher for a value of total OSA ranging between 3.4° and 8.9°. For a small value of total OSA (1.2°–2.2°), the USR is poor, even though the shape of the scan output appears to be close to a triangular waveform. In cases with small scan angles, the high-frequency noise induced an additional degradation of 1.5% in SLE, as opposed to cases with large scan angles. This observation can be generally explained by the fact that the same amount of noise would degrade a USR with a small total OSA more than it would that with a large total OSA because the tolerable scan error becomes stricter for a smaller total OSA. Note that the NRMSE value shows a tendency to decrease with the USR. This implies that USR is valid as an effective performance index of the optical microscanner.

### 4.2. Driving Frequency

To examine the application limits of ISETF with respect to the driving frequency, the ISETF was experimentally implemented for driving frequencies from 20 Hz to 300 Hz. Generally, the higher the sampling rate, the more frequency components are included in the process of input shaping, resulting in the desired output. Thus, the sampling rate was fixed at 50 kHz to avoid the influence of the sampling rate on the performance. The driving voltage was also determined to generate a constant scan angle of 5.6° in the total OSA, at which one of the best USRs was achieved in Section 4.1.

**Figure 10:**
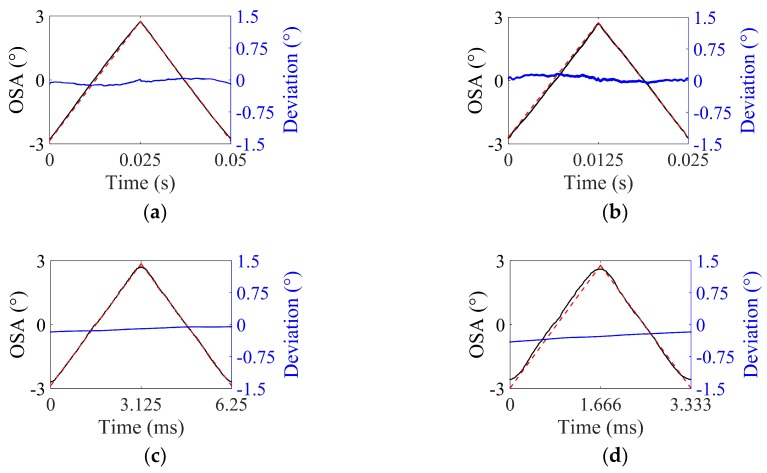
Performance of the ISETF with respect to the driving frequency (driving voltage: 25 V, sampling rate: 50 kHz): (**a**) 20 Hz, (**b**) 40 Hz, (**c**) 160 Hz, and (**d**) 300 Hz (scan output using the ISETF: solid line in black; ideal triangular output: dashed lines in red).

Figure 10 compares the experimental scan output (solid line in black) and the ideal triangular output (dashed line in red) for various driving frequencies. When the frequency increases, the experimental scan output tends to deviate from the ideal triangular output. Particularly, the driving signals at the high frequencies tend to excite the natural oscillation mode at 14.6 kHz, which do not have a large enough amplitude to degrade the scan output.

**Figure 11:**
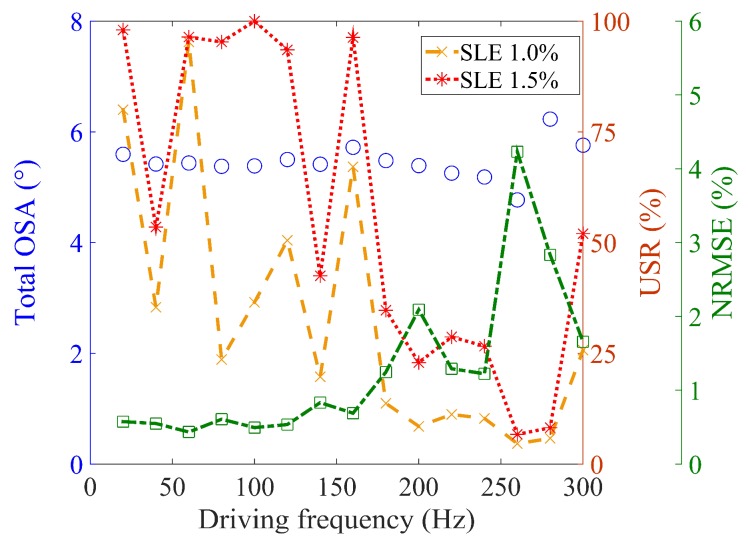
Experimental performance of the proposed input shaping with respect to driving frequency (driving voltage: 25 V, sampling rate: 50 kHz): total OSA with respect to driving frequency (circles in blue), usable scan range (dashed lines), and NRMSE (dash–dot line in green).

To quantitatively evaluate the scan outputs, the NRMSE and USR were analyzed with respect to the driving frequency, as shown in Figure 11. The total OSAs are also displayed, excluding the region with C-nonlinearity. The experimental total OSA varies slightly with respect to the driving frequency, despite the scanner being actuated by the driving voltage estimated to generate a constant total OSA value of 5.6°.

The NRMSE values remain below 1% for frequencies up to 160 Hz, which corresponds to the C-linear region for a scan angle of 5.6° or smaller. The NRMSE values abruptly degrade beyond the C-linear region.

The USR is 90% or higher for up to 160 Hz, except at two frequencies, when the SLE is 1.5%. To examine why the USR is specifically poor at the two frequencies of 40 Hz and 140 Hz, the frequency components of the triangular input were analyzed for the two driving frequencies and compared with the fundamental mode (264 Hz) of the microscanner. It was found that the fifth harmonic of 40 Hz and the first harmonic of 140 Hz are closer to the fundamental mode than those of other driving frequencies. These resonances are believed to be the major causes of the lower USR and larger NRMSE of the corresponding scan output.

There can be another possible explanation for the fact that the USRs are as poor as 55% or less, even if the shapes of the scan output are apparently close to the ideal triangular output. The poor USR might be partly attributed to the definition of USR that is determined by the region continuously satisfying the SLE requirement. In other words, there should be no single data point that deviates from the ideal triangular output beyond the SLE requirement. Indeed, although the USR is very practical as a performance index, it might sometimes lose its statistical meaning, unlike NRMSE.

For a driving frequency of 180 Hz or higher, the USRs fall off below 52% because the higher driving frequencies include a smaller number of harmonics in the input shaping calculation. The reduction in harmonics is attributed to the limitation from the Nyquist criterion, which results in the degradation of the triangular shape of the output. Note that the NRMSE value decreases with the USR, implying that the validity of the USR in the range of driving frequency is similar to that in the range of the OSA.

### 4.3. Comparison to Other Methods

As shown in Table 2, the performance of the ISETF is experimentally compared with those of other methods [9]; this paper contains the best results of input shaping for open-loop and closed-loop control. For a quantitative comparison of the proposed ISETF method to the conventional methods, the driving frequencies were normalized by the resonance frequencies of the scanners used in each experiment. Half of the angle error (peak-to-peak error) was also assumed to correspond to the NRMSE value.

Under these conditions, critical frequency can be defined based on relative superiority in performance. The normalized peak-to-peak error of the ISETF is slightly inferior to those of the open-loop and closed-loop control by 0.14% up to 40 Hz and 0.33% up to 155 Hz, respectively, in terms of average values; the normalized peak-to-peak error as a percentile are equivalent to 7.84 m° and 18.5 m° for the total OSA of 5.6°, respectively. For the driving frequencies from the critical frequencies up to 220 Hz, the normalized peak-to-peak error of the ISETF is smaller than those of the open-loop control and closed-loop control by 1.60% (89.6 m°) and 2.63% (147.3 m°), respectively, in terms of average values. The better performance of the ISETF is particularly revealed by the reduced normalized peak-to-peak error values: by 11.7% (655.2 m°) at 130 Hz and 6.1% (341.6 m°) at 220 Hz, respectively. This is attributed to the fact that the ISETF compensated both the fundamental mode and higher modes, whereas other control methods do not.

## 5. Conclusions

This method directly uses the experimental transfer function (ETF) with no dynamic modeling of the microscanner. The ISETF enables the ETF to include harmonics, higher modes, and/or sub-resonance so that the transfer function can be obtained more accurately than by the conventional open-loop control. The proposed ISETF method was confirmed to effectively remove residual oscillation caused both by the fundamental mode and by the higher modes of a microscanner. The limitations of the ISETF were experimentally examined with respect to the OSA and frequency, showing that a large USR was achievable for OSAs up to 8.9° and for driving frequencies in the range up to 160 Hz. From the experimental results, it is expected that the proposed ISETF can effectively reduce the residual oscillation caused by higher modes or crosstalk in two-axis driving compared to the conventional open-loop method. As a further study, electrodes with a larger thickness are being considered in experiments to extend the application of the ISETF to larger scan angles.

## Figures and Tables

**Table 1:** Dimensions of the comb electrodes in the microscanner.

Parameters	Values
Number of fingers per pair of electrodes, *N*	126
Thickness of the electrode, *t*	50 μm
Length of the electrode, *l_e_*	175 μm
Width of the electrode, *w_e_*	5 μm
Distance to the rotation axis, *l_r_*	475 μm
Electrode gap, *g*	5 μm

**Table 2:** Performance comparison between open-loop control and the proposed ISETF.

**Control method**		**Open-loop control [9]**	**This paper**
Frequency of fundamental torsional mode (FTM)		120 Hz	264 Hz
Total optical scan angle (total OSA)		20°	5.6°
20–40 Hz	Peak-to-peak error	66.0 m°	31.9 m°
	Normalized peak-to-peak error	**0.33 %**	**0.57%**
60–220 Hz	Peak-to-peak error	500.0 m°	50.4 m°
	Normalized peak-to-peak error	**2.50%**	**0.90%**
**Control method**		**Closed-loop control [9]**	**This paper**
20–140 Hz	Peak-to-peak error	46.0 m°	31.4 m°
	Normalized peak-to-peak error	**0.23 %**	**0.56%**
160–220 Hz	Peak-to-peak error	790.0 m°	73.9 m°
	Normalized peak-to-peak error	**3.95 %**	**1.32 %**

## Abbreviations

ETF	Experimental transfer function
ISETF	Input shaping method based on an experimental transfer function
NRMSE	Normalized root-mean-square error
OSA	Optical scan angle
SLE	Scan line error
USR	Usable scan range
